# A New Immunotherapy Combination Promises to Improve Survival for Patients with Metastatic Prostate Cancer

**DOI:** 10.3390/cancers15235640

**Published:** 2023-11-29

**Authors:** Juliette R. Seremak, Bal L. Lokeshwar

**Affiliations:** Georgia Cancer Center, Medical College of Georgia, Augusta University, Augusta, GA 30912, USA; juseremak@augusta.edu

## 1. Introduction

Prostate cancer (PC) is the second-most prevalent malignancy affecting the male population worldwide [1]. The global PC incidence in 2020 was estimated at 1.4 million new cases, with over 375,000 deaths [1]. The estimated number of new diagnoses for PC in the United States in 2023 is 288,300, and about 32,000 will die of this disease [2], making PC a prominent focus in cancer research and treatment advances. Metastatic castration-resistant prostate cancer (mCRPC) is a subtype of PC that frequently is the last (lethal) stage of the disease, accounting for about 90% of all deaths related to PC [3]. The mCRPC is a lethal disease in need of more efficacious treatment options, as indicated by the dismal median survival rate, which is less than two years [4]. A rare subset of mCRPC patients with liver metastasis have even fewer sustainable options for therapy and have a median overall survival (OS) of 13.5 months [5,6]. It is widely known that mCRPC is associated with a low tumor mutational burden (TMB) [7], suggesting that immunotherapies may be ineffective for this type of cancer. However, with vast improvements in technology over the last few decades and the emergence of precision medicine, a new emphasis on using immunotherapies has emerged as a promising approach for effectively combating mCRPC [8], including exploration of the use of various immunomodulatory combinations that incorporate vaccine-based therapies, immune checkpoint inhibitors (ICIs), and chimeric antigen receptor (CAR) T cells [9]. 

Recent studies have shown that immunotherapy can enhance the immune system’s ability to recognize and target cancer cells, leading to improved clinical outcomes in patients with mCRPC [10]. Vaccine-based therapies, such as sipuleucel-T, have been shown to improve OS by approximately 13 months in mCRPC patients [11,12]. However, sipuleucel-T, or Provenge, is only available to patients who are asymptomatic or have minimal symptoms, further limiting options for patients who are symptomatic or have liver metastasis [13]. ICIs, such as pembrolizumab and nivolumab, have also demonstrated efficacy, particularly in patients with high tumor mutational burden [7]. CAR T cells, which are genetically engineered to target specific antigens on cancer cells, have shown promising results in preclinical studies [14]. Currently, there are ongoing clinical trials testing various combinations with ICIs to improve the efficacy of current treatment strategies for mCRPC. Here, we highlight two reviews by Venkatachalam et al. [13] and Bansal et al. [14], focusing on preclinical findings and current ongoing trials using single-agent immunotherapies and ICIs in combination with other available options to evaluate potentially more efficacious treatment options for patients with mCRPC.

## 2. Unlocking the Potential of ICIs

Venkatachalam et al.’s [13] article focuses specifically on the use of ICIs in PCs. The authors provide an overview of the immune checkpoint pathways involved in PC and discuss the rationale for targeting these pathways with checkpoint inhibitors, emphasizing PD-1 and PD-L1 expression in mCRPC. The authors then review the clinical data on ICIs in PC, including the results of recent trials investigating the use of these agents in combination with other agents, such as androgen or poly ADP-ribose polymerase (PARP) inhibitors, different types of radio- or cryotherapies, tumor vaccines (i.e., sipuleucel-T), chemotherapy drugs, tyrosine kinase inhibitors, and other combinations involving granulocyte-macrophage colony-stimulating factor (GM-CSF). The authors also report whether prostate-specific antigen (PSA) serum levels declined as an indicator of treatment efficacy, as elevated serum PSA levels are associated with mCRPC [15]. Lastly, Venkatachalam et al. [13] also discuss the challenges associated with the use of ICIs in PC, including the importance of identifying biomarkers to select patients who are most likely to benefit from treatment with immunomodulatory agents. Overall, the future direction of mCRPC treatment depends on the outcome of the current phase 1 and phase 2 clinical trials. 

## 3. Using Immunotherapy Combinations to Improve mCRPC Treatment Efficacy

In Bansal et al.’s review on the use of immunotherapy combinations to enhance treatment in mCRPC [14] the authors evaluate various promising studies and ongoing trials to provide a detailed overview of the current treatment landscape for castration-resistant prostate cancers, including the use of vaccine-based therapies categorized into four distinct types (i.e., DNA-, peptide-, viral vector-, and cell-based vaccines) and ICIs, such as CTLA-4 and PD-1 inhibitors, along with highlighting the limitations of using existing monotherapies. Since PC is typically associated with a low TMB, this review aims to discuss combination therapies with currently available treatment options for mCRPC, which could potentially transform “cold” tumors into “hot” tumors in a synergistic manner to enhance responses in patients. Similarly to Venkatachalam et al.’s review [13], the authors also discuss current clinical trials investigating the use of immunotherapy combinations in mCRPC, including vaccine-based therapies, ICIs, such as chimeric antigen receptor (CAR) T cells, GM-CSF, PARP inhibitors, and chemotherapy agents in novel combinations, and provide insights into the future direction of research in this area to achieve an immune response for immunologically “cold” cancers like PC. For concluding remarks, the authors again stress the emphasis that the key to improving current therapies is by better understanding tumor biology and recognizing predictive biomarkers to determine successful outcomes for patients and further personalize treatments as technology continues to advance.

## 4. Conclusions

Overall, both articles provide valuable insights into the use of immunotherapy in prostate cancer and highlight the potential of these therapies to improve outcomes for patients with lethally advanced PC. The articles also underscore the need for further research on the tumor microenvironment (TME) to optimize the use of immunotherapy in this setting and to identify biomarkers that can predict response to treatment when selecting therapies for patients. Taken together, immunotherapy and combination immunomodulatory strategies incorporating vaccine-based therapies, ICIs, and CAR T cells represent a promising approach for the treatment of mCRPC. However, further research and, more importantly, clinical trials are needed to optimize these therapies and improve clinical outcomes for patients with this disease, along with the need for scientists and clinicians to collaborate to design highly valuable clinical trials using novel therapies. Regardless of these requirements, the future seems to point toward accelerated development for treating this dreaded disease.
